# Cardiac hemodynamic response to the 6-minute walk test in young adults and the elderly

**DOI:** 10.1186/s13104-015-1331-5

**Published:** 2015-08-18

**Authors:** Fujiko Someya, Naoki Mugii, Sachie Oohata

**Affiliations:** School of Health Sciences, Kanazawa University, Kodatsuno 5-11-80, Kanazawa, 920-0942 Japan; Division of Rehabilitation, Kanazawa University Hospital, Kanazawa University, Takaramachi 13-1, Kanazawa, 920-8641 Japan

**Keywords:** Impedance cardiography, Stroke volume, Cardiac output, Distance walked, Heart rate

## Abstract

**Background:**

Exercise capacity is evaluated using the 6-minute walk test in various diseases. Variety in the distances walked was also shown in healthy subjects. Moreover, age-related influences on cardiac hemodynamic response to the 6-minute walk test have not been clarified. The purpose of this study was thus to investigate the hemodynamic response to the 6-minute walk test and to detect factors related to the distance walked in healthy subjects.

**Methods:**

Thirteen young adults (age 20.5 ± 0.7 years, BMI 22.0 ± 4.3) and 26 elderly individuals (age 60.2 ± 6.1 years, BMI 21.7 ± 2.2) were enrolled to measure real-time hemodynamic responses using non-invasive impedance cardiography during the 6-minute walk test.

**Results:**

Stroke volume was higher in the young than in the elderly and reached a plateau within 30 s of starting to walk in all subjects. An increase in heart rate took more than 1 min in the elderly, while it took less than 30 s in the young, which resulted in slower increases in cardiac output and cardiac index in the elderly. There was no difference in the distance in the 6-minute walk test between the young and the elderly. The distance walked was correlated with heart rate, cardiac output, and cardiac index, but not with stroke volume, at the end of the 6-minute walk test.

**Conclusions:**

The distance walked appeared to depend on increased cardiac output based on heart rate, but did not appear to be limited by stroke volume, in healthy subjects.

## Background

Exercise capacity has predominantly been evaluated by cardiopulmonary exercise or the 6-minute walk test (6MWT). The 6MWT does not require expensive equipment and is easily utilized for various patient populations. Guidelines for the 6MWT have been published by the American Thoracic Society [1], and high reproducibility of the 6MWT was confirmed [2, 3]. However, geographic variations in the distances walked were also noted in healthy adults and a wide range of exercise intensities calculated by heart rate during the 6MWT were reported from 59 to 88 % as percentage of maximal heart rate [4], which made it difficult to apply predictive equations using age, height and weight for the distance walked presented by Enright and Sherrill [5]. Moreover, the longitudinal improvement of distance in the 6MWT varied according to studies from 25 m [6] to 70 m [7] in patients with COPD. Thus, a rational explanation for the variety of distances in 6MWT in healthy adults is needed.

Hemodynamic responses have been examined using non-invasive impedance cardiography during cardiopulmonary exercise testing [8]. A linear relationship between oxygen uptake and cardiac output or heart rate during cardiopulmonary exercise was shown, whereas maximal stroke volume was reached at submaximal intensities below the anaerobic threshold [9]. Additionally, it is known that age is negatively associated with oxygen uptake, cardiac output, and heart rate at peak exercise [10, 11].

The 6MWT is a submaximal exercise test used to evaluate exercise capacity in individuals with a wide range of ages [4]; however, hemodynamic response during the 6MWT was not clarified. The purpose of this study was to detect hemodynamic factors to explain the variety of distances walked.

## Methods

Thirty-nine healthy adults (29 females and 10 males) were enrolled in this study. Thirteen of them were young (age 20.5 ± 0.7 years, BMI 22.0 ± 4.3, expressed as mean ± SD) and 26 were elderly (age 60.2 ± 6.1 years, BMI 21.7 ± 2.2) (Table 1). No subjects had previously performed the 6MWT, which was reported to increase the distance walked [1]. The young were university students volunteering for this study. Eighteen of the elderly were recruited from among university hospital staff and 8 of them were from among 42 voluntary participants of a walking class held twice a week for residents living near our university. Exclusion criteria were experience of heart disease, central nervous system disease, or musculoskeletal disorders, or receiving β-blocker medication. The protocol of the study was approved by the Human Ethics Committee of Kanazawa University according to the principles expressed in the Declaration of Helsinki, and all subjects gave their written informed consent to participate in this study.

**Table 1:** Characteristics of the subjects (n = 39)

	Young	Elderly	P
Subject number (f/m)	8/5	21/5	0.25
Age (years)	20.5 ± 0.7 (20–22)	60.2 ± 6.1 (48–74)	<0.001
Height (cm)	166 ± 2 (148–183)	160 ± 8 (147–179)	0.04
Weight (kg)	61 ± 17 (38–110)	56 ± 8 (37–73)	0.26
Body mass index (kg/m^2^)	22.0 ± 4.3 (17.3–34.3)	21.7 ± 2.2 (16.9–25.3)	0.80
6-minute walk distance (m)	541 ± 46 (475–620)	533 ± 78 (430–743)	0.68
Exercise intensity (%)	59 ± 13 (37–92)	76 ± 14 (53–102)	0.001

Values are expressed as mean ± standard deviation. Numbers in parentheses represent ranges of values. P values between young and elderly subjects

The 6MWT was performed by the subjects following Guidelines by the American Thoracic Society [1]. All subjects were tested by the same trained technician. Hemodynamic responses were measured using the PhysioFlow Q-Link (Manatec Biomedical, France) weighing 200 g, and non-invasive impedance cardiography was undertaken using a tablet, Surface Pro 2 (Microsoft Corporation, USA), loading the impedance software. Six disposable electrodes, Blue Sensor T (Ambu, Denmark), were placed on the subjects: two pairs of a transmitting electrode and a sensing electrode on the left neck and at the xyphoid area, V1 and V6 positions, to monitor the ECG signal [12]. After autocalibration for 30 s of rest, the participants carried the equipment in a small bag with a shoulder belt during the 6MWT. One minute of recovery time was added to the 6MWT in a standing still posture at the place where the subjects stopped walking. Stroke volume, heart rate, cardiac output, and cardiac index were averaged every 10 s. The data at rest, 30 s, 1, 2, and 6 min of walking, and 1 min of recovery were collected for analyses. The exercise intensity at the end of the 6MWT was calculated using a formula (heart rate at 6 min/estimated maximal heart rate), setting the estimated maximal heart rate as (220 − age).

### Statistical analyses

Comparison of the gender distribution was performed by Chi squared test. Unpaired t tests were used to determine age-related differences in the measured values on height, weight, body mass index, the distance walked, and the exercise intensity. Two-way analyses of variance were used to determine age-related differences and walked time-related differences in values of stroke volume, heart rate, cardiac output, and cardiac index. If time-related difference was shown, each value of the four cardiac responses at 6 min was compared to those at other evaluation times using Dunnett’s test to show the time taken for each parameter to reach a plateau. The relationships between the distance walked and stroke volume, heart rate, cardiac output, cardiac index, and exercise intensity at 6 min of the test were determined by linear regression (Pearson’s). JMP 8 software (SAS Institute Inc., Cary, NC, USA) was used for statistical analysis. P < 0.05 was considered statistically significant.

## Results

There were no differences between the young and the elderly in terms of gender, weight, and body mass index, but the young were taller (Table 1). The distance walked did not show a significant difference in terms of age, whereas exercise intensity of the 6MWT was significantly higher in the elderly than in the young (p = 0.001). Exercise intensity was above 50 %, except in two young subjects, and was widely distributed in the young and the elderly.

**Fig. 1:**
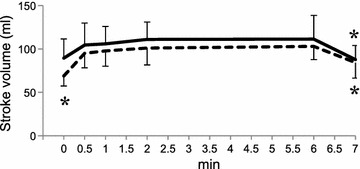
Stroke volume response to the 6-minute walk test and additional recovery time of 1 min. Data are expressed as mean ± SD. *p < 0.05 versus value at 6 min by Dunnett’s test for the young (*solid line*) or the elderly (*dotted line*)

**Fig. 2:**
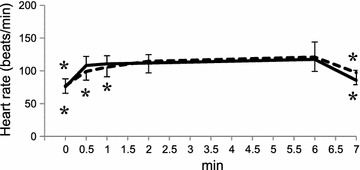
Heart rate response to the 6-minute walk test and additional recovery time of 1 min. Data are expressed as mean ± SD. *p < 0.05 versus value at 6 min by Dunnett’s test for the young (*solid line*) or the elderly (*dotted line*)

**Fig. 3:**
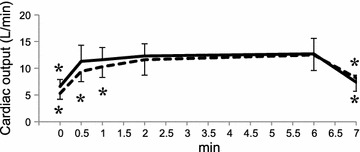
Cardiac output response to the 6-minute walk test and additional recovery time of 1 min. Data are expressed as mean ± SD. *p < 0.05 versus value at 6 min by Dunnett’s test for the young (*solid line*) or the elderly (*dotted line*)

**Fig. 4:**
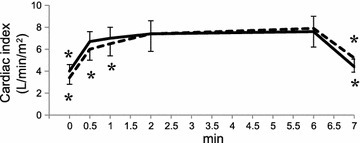
Cardiac index response to the 6-minute walk test and additional recovery time of 1 min. Data are expressed as mean ± SD. *p < 0.05 versus value at 6 min by Dunnett’s test for the young (*solid line*) or the elderly (*dotted line*)

Stroke volume in the young was larger than in the elderly (p = 0.001; Table 2). The increase of stroke volume in the young was not significant during 6MWT; however, a rapid increase within 30 s was observed in the elderly and the last stroke volume of the 6MWT reached 102.9 ± 15.2 ml, while it was 111.3 ± 27.3 ml in the young (Fig. 1; Table 2). In heart rate, there was no difference between the young and the elderly at rest and at 6 min (p = 0.56; Table 2). During the 6MWT, heart rate increased at the beginning of the 6MWT in the young and reached a plateau within 30 s, while this took over 1 min in the elderly (Fig. 2). The slower increase in cardiac output and cardiac index during the 6MWT was also observed in the elderly (Figs. 3, 4). There were no significant age-related differences in cardiac output and cardiac index at rest and at 6 min (p = 0.14 and 0.65, respectively; Table 2). All of the hemodynamic parameters decreased after the recovery time of 1 min, and a tendency to decrease slowly in heart rate and cardiac index was also observed in the elderly.

**Table 2:** Cardiac hemodynamic response to the 6-minute walk test

	Young	Elderly	P		
			Time	Age	Time × age
Stroke volume (ml)					
Rest	89.4 ± 22.1	68.9 ± 11.6	<0.001	0.001	0.17
6 min	111.3 ± 27.3	102.9 ± 15.2			
Heart rate (beats/min)					
Rest	75.8 ± 12.0	77.3 ± 11.6	<0.001	0.56	0.82
6 min	117.4 ± 26.7	121.0 ± 21.8			
Cardiac output (l/min)					
Rest	6.6 ± 1.3	5.3 ± 1.1	<0.001	0.14	0.34
6 min	12.7 ± 2.9	12.5 ± 2.9			
Cardiac index (l/min/m^2^)					
Rest	4.0 ± 0.6	3.4 ± 0.6	<0.001	0.65	0.12
6 min	7.6 ± 1.4	7.9 ± 1.7			

Values are expressed as mean ± standard deviation. P values in time are between rest and 6 min, and in age are between young and elderly subjects

**Fig. 5:**
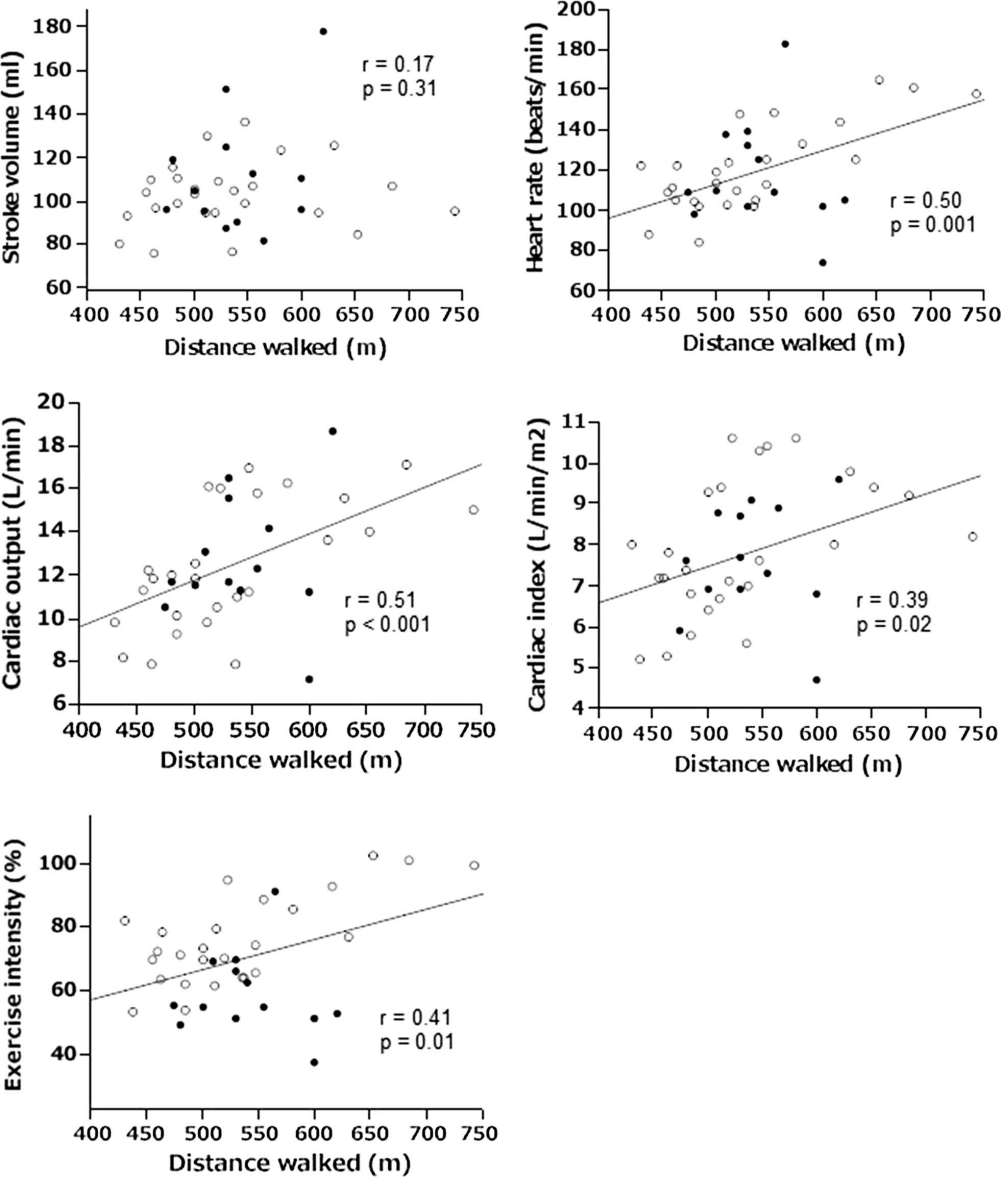
Correlations between the distance walked and cardiac hemodynamic responses. The young are represented by *filled circles* and the elderly by *open circles*. The regression line for all subjects was inserted when p < 0.05

The distance walked during the 6MWT was not correlated with stroke volume at 6 min of the 6MWT (Fig. 5). The distance walked was positively correlated with heart rate, cardiac output, and cardiac index at 6 min of the 6MWT (p = 0.001, <0.001, and 0.02, respectively). The distance walked was also positively correlated to exercise intensity (p = 0.01).

## Discussion

The distance walked during the 6MWT appeared to depend on the obtained cardiac output based on heart rate or exercise intensity, but not on age, in the present study. However previous studies showed the distance walked correlated with age [4, 5], the American Thoracic Society mentioned that the self-paced 6MWT assessed the submaximal level of functional capacity and the subjects chose their own intensity of exercise [1]. The exercise intensity of the elderly in this study was significantly higher than that of the young, despite the same instructions in the test by the same researcher. Although the reason for the age-related difference in exercise intensity could not be fully explained, the distance walked by the elderly being the same as that by the young seemed to be due to the intensity that the subjects chose.

Stroke volume in the elderly rapidly increased and reached a plateau within 30 s of walking. As stroke volume in the young at rest seemed to be sufficient to undergo the 6MWT, it did not change during the test. Previously, stroke volume was shown to increase within the range of 50 % of maximal work output during incremental cardiopulmonary exercise testing in healthy elderly of over 60 years of age [9]. Since exercise intensity was above 50 % in most of the subjects, it is suggested that the maximal stroke volume could be shown by the 6MWT in healthy adults.

A conflicting study showed that stroke volume at maximal exercise was higher than that at the 6MWT in healthy subjects of around 40 years of age [13]. However, only 7 healthy subjects were studied and the difference in stroke volume between 6MWT and maximal exercise was small in some subjects. Moreover, Ferreira et al. [14] demonstrated three different patterns of stroke volume response during progressive exercise in patients with pulmonary arterial hypertension. Further examination of stroke volume response during the 6MWT in various patients should be undertaken.

For example, stroke volume in patients with chronic cardiac failure [15] or pulmonary arterial hypertension [13, 14] did not increase as much as in healthy subjects at peak exercise. In particular, an increase of less than 10 ml in stroke volume during cardiopulmonary exercise testing was significantly associated with less than 50 % of predicted peak oxygen uptake [14]. These studies implied that certain impairment of exercise capacity could be demonstrated by insufficient stroke volume during the 6MWT compared with that in healthy subjects.

In the present study, stroke volume in the young was larger than in the elderly, however previous findings regarding the effect of healthy aging on stroke volume has been conflicting, like to be unaffected [10, 16]. Methodological differences or measurement techniques were suggested to contribute to the discrepancies in previous findings [10], and there were few age-related studies using non-invasive impedance cardiography.

Heart rate was still increasing when stroke volume reached a plateau, so cardiac output and cardiac index increased in proportion to heart rate. Additionally, our findings showed that the increase in heart rate in the elderly was slower than in the young, although heart rate reached a plateau within 2 min of the test on average. The age-related difference in the dynamics of heart rate is well known, and the age-related reduction in sympathetic activation is the likely reason for the slower heart rate kinetics [17]. However, the slower response in the elderly did not seem to reduce the distance walked because the last heart rate in the 6MWT was significantly correlated to the distance walked in all subjects.

We do not deny the importance of the distance walked during the 6MWT for the evaluation of exercise capacity, although the distance walked could vary depending on the individual’s exercise intensity, even in healthy people. A value of <350 m of the distance walked was associated with increased mortality in 1379 patients with chronic obstructive pulmonary disease [18]. In addition, the distance walked during the 6MWT was utilized for representing the long-term course of disease and the effects of treatment [19]. Hence, it was suggested that the 6MWT could be a good test for assessing exercise capacity, especially representing the individual clinical course, which is also due to the high reproducibility of the distance walked [2, 3]. We would just like to emphasize that individual variation of the exercise intensity for the 6MWT cannot be avoided. This probably implies that the extremely precise comparison of differences in the distance walked might be useless in the cross-sectional study.

There are some limitations in this study. The number of the young was smaller than that of the elderly. However, the range of age in the young was narrow, at 2 years, while it was 26 years in the elderly. It might be suggested that the evaluation values in the young could represent the age-specific response by means of a small number of subjects. Moreover, as the young subjects were students at our university, they were familiar with the researchers, while the elderly were not known to the researchers at the time of the evaluation. This difference in familiarity could have influenced the individuals’ exercise intensity. Even though the difference in the exercise intensity was considered, the intensity was widely distributed in both the young and the elderly, and an age-related difference in stroke volume was clearly shown. This may imply that the 6MWT could be utilized to evaluate hemodynamic responses regardless of the variety of exercise intensities during walking.

## Conclusions

The distance walked during the 6MWT depended on the exercise intensity or cardiac output based on heart rate, although the heart rate response in the elderly was slower than in the young. It should be noted that stroke volume during the 6MWT was not influenced by exercise intensity, reached a plateau within 30 s of starting to walk, and did not seem to affect the distance walked in healthy subjects.

## References

[1] American Thoracic Society (2002). ATS statement: guidelines for the six-minute walk test. Am J Respir Crit Care Med.

[2] Bellet RN, Francis RL, Jacob JS, Healy KM, Bartlett HJ, Adams L (2011). Repeated six-minute walk tests for outcome measurement and exercise prescription in outpatient cardiac rehabilitation: a longitudinal study. Arch Phys Med Rehabil.

[3] Buch MH, Denton CP, Furst DE, Guillevin L, Rubin LJ, Wells AU (2007). Submaximal exercise testing in the assessment of interstitial lung disease secondary to systemic sclerosis: reproducibility and correlations of the 6-min walk test. Ann Rheum Dis.

[4] Casanova C, Celli BR, Barria P, Casas A, Cote C, de Torres JP (2011). The 6-min walk distance in healthy subjects: reference standards from seven countries. Eur Respir J.

[5] Enright PL, Sherrill DL (1998). Reference equations for the six-minute walk in healthy adults. Am J Respir Crit Care Med.

[6] Holland AE, Hill CJ, Rasekaba T, Lee A, Naughton MT, McDonald CF (2010). Updating the minimal important difference for six-minute walk distance in patients with chronic obstructive pulmonary disease. Arch Phys Med Rehabil.

[7] Redelmeier DA, Bayoumi AM, Goldstein RS, Guyatt GH (1997). Interpreting small differences in functional status: the six minute walk test in chronic lung disease patients. Am J Respir Crit Care Med.

[8] Richard R, Lonsdorfer-Wolf E, Charloux A, Doutreleau S, Buchheit M, Oswald-Mammosser (2001). Non-invasive cardiac output evaluation during a maximal progressive exercise test, using a new impedance cardiograph device. Eur J Appl Physiol.

[9] Farinatti PT, Soares PP (2009). Cardiac output and oxygen uptake relationship during physical effort in men and women over 60 years old. Eur J Appl Physiol.

[10] Carrik-Ranson G, Hastings JL, Bhella PS, Shibata S, Fujimoto N, Palmer D (2013). The effect of age-related differences in body size and composition on cardiovascular determinants of VO_2max_. J Gerontol A Biol Sci Med Sci.

[11] van Empel VPM, Kaye DM, Borlaug BA (2014). Effects of healthy aging on the cardiopulmonary hemodynamic response to exercise. Am J Cardiol.

[12] Tonelli AR, Alnuaimat H, Li N, Carrie R, Mubarak KK (2011). Value of impedance cardiography in patients studied for pulmonary hypertension. Lung.

[13] Deboeck G, Taboada D, Hagan G, Treacy C, Page K, Sheares K (2014). Maximal cardiac output determines 6 minutes walking distance in pulmonary hypertension. PLoS One.

[14] Ferreira EM, Ota-Arakaki JS, Barbosa PB, Siqueira ACB, Bravo DM, Kapins CEB (2012). Signal-morphology impedance cardiography during incremental cardiopulmonary exercise testing in pulmonary arterial hypertension. Clin Physiol Funct Imaging.

[15] Fukuda T, Matsumoto A, Kurano M, Takano H, Iida H, Morita T (2012). Cardiac output response to exercise in chronic cardiac failure patients: role of stroke volume. Int Heart J.

[16] Rowland T, Popowski B, Ferrone L (1997). Cardiac responses to maximal upright cycle exercise in healthy boys and men. Med Sci Sports Exerc.

[17] Fukuoka Y, Nakagawa Y, Ogoh K, Shiojiri T, Fukuba Y (2002). Dynamics of the heart rate response to sinusoidal work in humans: influence of physical activity and age. Clin Sci.

[18] Cote CG, Casanova C, Marin JM, Lopez MV, Pinto-Plata V, de Oca MM (2008). Validation and comparison of reference equations for the 6-min walk distance test. Eur Respir J.

[19] Foglio K, Bianchi L, Bruletti G, Porta R, Vitacca M, Balbi B (2007). Seven-year time course of lung function, symptoms, health-related quality of life, and exercise tolerance in COPD patients undergoing pulmonary rehabilitation programs. Respir Med.

